# Does it matter for health if steps are taken during work or leisure? A prospective accelerometer study using register-based long-term sickness absence

**DOI:** 10.1186/s12966-023-01468-4

**Published:** 2023-06-09

**Authors:** Marie Raunkjær Christensen, Kirsten Nabe-Nielsen, Andreas Holtermann, Nidhi Gupta

**Affiliations:** 1 The National Research Centre for the Working Environment, Lersø Parkallé 105, Copenhagen Ø, 2100 Denmark; 2 Department of Public Health, Section of Social Medicine, University of Copenhagen, Copenhagen, Denmark; 3 Department of Sports Science and Clinical Biomechanics, University of Southern Denmark, Odense, Denmark

**Keywords:** Walking, Sick leave, Absenteeism, Work demands, Occupational health, Physical activity paradox

## Abstract

**Background:**

Walking is known to be good for health. However, it is unknown whether it matters if steps are taken during work or leisure. Therefore, we aimed to examine the prospective association between accelerometer-measured steps taken during work or leisure and register-based long-term sickness absence (LTSA).

**Methods:**

We included 937 blue- and white-collar workers from the PODESA cohort who wore a thigh-based accelerometer over four days to measure number of steps during work and leisure. Steps were divided into domain based on diary data. First event of LTSA was retrieved from a national register with four years’ follow-up. We used Cox proportional hazard models to analyze the association between domain-specific and total daily steps and LTSA, adjusted for age, sex, job type, smoking, and steps in the other domain (e.g., work/leisure).

**Results:**

We found more steps at work to be associated with a higher LTSA risk [Hazard Ratio (HR):1.04; 95% CI: 1.00–1.08 per 1000 steps]. No significant association was found between steps during leisure and LTSA (HR: 0.97; 95% CI: 0.91–1.02), nor between total daily steps and LTSA (HR: 1.01; CI 95% 0.99–1.04).

**Conclusions:**

More steps at work were associated with higher risk of LTSA, while steps during leisure was not clearly associated with LTSA risk. These findings partly support ‘the physical activity paradox’ stating that the association between physical activity and health depends on the domain.

**Supplementary Information:**

The online version contains supplementary material available at 10.1186/s12966-023-01468-4.

## Background

The physical and mental health benefits of walking many steps per day are well-established, as number of steps walked per day is inversely associated with the risk of all-cause mortality, cardio-vascular morbidity, and depression [1–4].

Number of steps are also shown to be associated with important work-related outcomes, such as lower risk of sickness absence [5, 6]. Sickness absence, especially long-term, is a considerable risk factor for permanent exclusion from the labor market [7–9], and is a large public health problem [7, 10, 11].

Previous studies have found that more physical activity conducted at work is associated with a higher risk of adverse outcome for health including sickness absence, while physical activity during leisure is associated with better health outcomes and lower sickness absence [12–14]. This intriguing opposite effect of physical activity in different domains is also known as ‘the physical activity paradox’ [15].

Only a few previous studies have examined the association between walking and sickness absence [5, 6, 16, 17]. Some studies found that walking (e.g. brisk walks or walking one bus stop) was associated with a lower risk of sickness absence [5, 6]. However, none of the previous studies measured number of steps, but solely duration of ‘walking’ [5, 6, 16, 17]. Moreover, all studies relied on self-reported data on physical activity and walking, known to be inaccurate and potentially biased [18, 19]. Additionally, these studies did not differentiate between walking during work and leisure [5, 6, 16, 17]. Thus, we lack knowledge on whether it matters for risk of sickness absence if steps are taken at work or during leisure. Knowledge about the association between walking during work and leisure on sickness absence risk is important to increase the workplace awareness of how walking at work may affect risk of sickness absence, and it is crucial for the design of healthy workplaces with better advice on health and prevention of sickness absence. Measuring number of steps is an easier way to track physical activity levels, than measuring duration of walking. Setting a goal to reach a certain number of steps per day is both easy with a modern devise (wristband or phone) and is a motivating and effective way to increase physical activity levels [20, 21]. Therefore, the aim of our study was to examine the prospective association between accelerometer-measured steps taken during work and leisure and register-based long-term sickness absence.

Our study, similar to Gupta et al. [12], explores the relationship between physical activity and long-term sickness absence in the same cohort population. However, while the previous study by Gupta et al. investigates *time* spent on physical activity, we contribute to the existing literature by focusing on the influence of *number* of steps, a widely accessible and measurable physical activity in both leisure and work domains, which has not been investigated previously. Our findings provide valuable insights into how steps influence long-term sickness absence and can be informative for both researchers and laypeople.

## Methods

### Data design and study population

In this four-year prospective study, workers participated in baseline measurements including accelerometry measurements, a questionnaire, and a health check. At baseline, workers’ step count was collected by thigh-worn accelerometers for up to four consecutive days. Characteristics of the workers were also collected. Participants were followed in a national register for four years from baseline to obtain information about events of long-term sickness absence. We selected a follow-up period that allowed us to capture a sufficient number of events for the statistical analysis while minimizing the influence of changes in jobs or exposures (e.g. steps during work and leisure) during follow-up.

This study is based on prospective data from the Danish ‘Physical wOrk DEmands and prospective register-based Sickness Absence study’ (PODESA) cohort [22]. The PODESA cohort comprises data from two cohort studies: The ‘Danish Physical activity cohort with objective measurements’ (DPhacto) and ‘New method for Objective Measurements of physical Activity in Daily living’ (NOMAD) [22].

The workplaces, which took part in DPhacto and NOMAD, were recruited in collaboration with the labor union representatives or health and safety representatives at the workplaces. To be included, the workplaces had to allow the workers to participate in the study activities during paid working hours [23]. Recruited workers came from 22 workplaces within sectors of construction, cleaning, transport, garbage, manufacturing, assembly work, mobile plant operator, health service and manufacturing.

The baseline data in the NOMAD and DPhacto cohorts were collected in 2011–2012, and 2012–2013 respectively. NOMAD and DPhacto used similar procedures for data collection and comprised predominantly blue-collar workers in Denmark, enabling easy harmonization [22].

For inclusion, workers had to be between 18 and 65 of age and work at least 20 h per week. Workers were excluded if they were pregnant, or reported allergy to bandage or adhesives. Previous studies did not find a clear difference between participants and non-participants in the NOMAD cohort [24, 25]. Details on the recruitment, data collection, inclusion, and exclusion criteria as well as the data-merging strategy for the construction of the PODESA cohort is described in detail elsewhere [22, 23, 26].

### Accelerometry-measured number of steps

The accelerometers used were of the model ActiGraph GT3X + (Florida, USA). These accelerometers are small, wireless, lightweight, waterproof sensors, which contain tri-axial accelerometers that can measure human postures and movements such as steps [27]. During the measurement, workers completed a daily diary reporting their time starting and ending work, going to and getting out of bed as well as non-wear periods. Data from the ActiGraph accelerometer recordings were first downloaded using the original commercial software of the equipment (ActiLife Software version 5.5) and further processed using a MATLAB software program Acti4 (The National Research Centre for the Working Environment, Copenhagen Denmark) for estimating duration of types of physical activity like walking [27]. Acti4 software has previously demonstrated high sensitivity and specificity in identifying physical activity in semi-standardized and full free-living settings during both work and leisure time [27, 28].

At least one workday including valid objective measurements during work and leisure per participant was required for data analyses. A leisure or work domain was considered valid if it comprised ≥ 4 h of wear time, or ≥ 75% of the individual’s average wear time across a day [12, 24]. These criteria are in line with previous studies that also used at least one valid day of measurements for estimating physical activities [29, 30]. The criteria have not been validated, but represents a tradeoff between 1) requirement of sufficient data (hours per day and number of days) to be representative for the physical activity of the participant (internal validity), and 2) not introducing bias by excluding participants who either work few hours per day or did not wear the accelerometer for the complete measurement period (external validity).

Total daily steps was defined by taking the average of steps taken during all valid measured days. The number of steps at work and during leisure was defined by taking the average of steps on all valid work and leisure periods during the four days of measurements. The information from participants’ diaries was used to define the work and leisure periods; a work period was from start till end of the work, and a leisure period was the remaining time of the day.

### Register-based data on long-term sickness absence

The data on long-term sickness absence were retrieved from the Danish Register for Evaluation of Marginalization (DREAM). The DREAM register contains weekly information on social transfer payments for all residents in Denmark, including weekly information on granted subsidized sickness absence for all individuals in Denmark and reimbursement of sickness pay [31, 32]. Long-term sickness absence was defined as the occurrence of the first event of ≥ 6 consecutive weeks of sickness absence, during the four year follow-up from the last day of accelerometry measurements at baseline. Consequently, each worker had equal follow-up time of 212 weeks (about four years). The cut-off point of 6 weeks of sickness absence was in accordance with previous research [33]. The data on sickness absence benefit from the DREAM register have shown excellent accuracy when compared with companies own records of employees’ sickness absence [31].

### Covariates

**Fig. 1:**
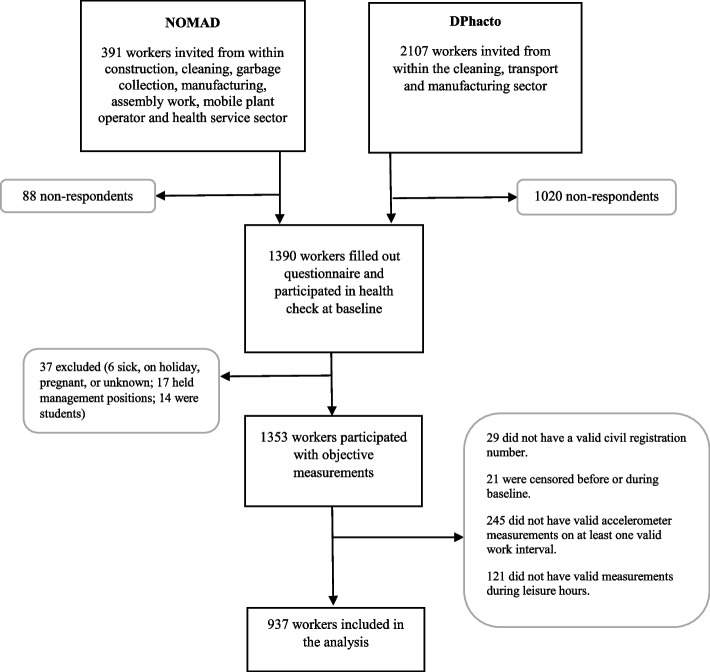
Flow of the participants in the study

Potential confounders where chosen based on prior related studies as well as literature on their association with the exposure and the outcome illustrated by Directed Acyclic Graphs (See Fig. 1A in the Additional file 1). Potential confounders were sex, age, smoking, job type and occupational lifting and carrying duration measured at baseline. Sex was determined using a single item. Age was determined using workers’ unique civil registration number. Smoking was determined using a single item in the baseline questionnaire with responses categorized into smokers (“smoking daily” or “sometimes”) or non-smokers (“ex-smokers” and “never smoked”). Duration of occupational lifting and carrying was determined using a single item “How much of your working time do you spend carrying or lifting things” with response categories “almost all the time”, “approximately ¾ of the time”, “approximately ½ of the time”, “approximately ¼ of the time”, “rarely or never”. Information about participants’ job type was collected through a single item: Are you an employee engaged in administrative work tasks (white collar) or in production (blue collar)?

### Statistical analyses

SPSS statistics (IBM, Version 27) was used for all statistical analyses.

To present the baseline characteristics of the study population according to the exposure to total daily steps, the study population was divided into four groups based on quartiles (Q) of total daily steps (Q1: < 9,472, Q2: 9,472–12,496, Q3: 12,497–16,473, Q4: > 16,473).

The Cox proportional hazard model was used to analyze the association between steps and long-term sickness absence, with results presented as hazard ratios (HR) per 1000 steps with their 95% confidence intervals (CI). In all analyses, the level of significance was *P* < 0.05 (5%). Cases with missing values were deleted list-wise. Consequently, number of participants differed between the analyses.

In the Cox proportional hazard model each participant contributed with risk time until their first event of long-term sickness absence or until the end of the four year follow-up in case of no events. 47 workers (5.0%) were censored during follow up, due to one of the following reasons: Emigration, death, entering early retirement, entering ordinary retirement, or becoming pregnant. Still, these participants contributed to the risk time in the analyses, until the week they were censored.

Steps during leisure, steps at work and total daily steps were examined separately. For all analyses, one crude and two adjusted models were applied. The crude model analyzed the association between steps (steps at work, steps during leisure and total daily steps, respectively) and long-term sickness absence, without adjusting for confounders. In model 1, adjustments were made for age and sex. In model 2 adjustments were made for age, sex, smoking, and job type. Furthermore, in the domain specific analysis of steps, steps at work and steps during leisure were mutually adjusted. Occupational lifting/carrying duration was considered a confounder. However, due to multi-collinearity between occupational lifting/carrying and job type (Pearson’s chi square test for correlation: X^2^ = 270.32, df = 5, *p* =  > 0.0001), this potential confounder was solely included in a sensitivity analysis.

Model assumptions related to the application of the Cox proportional hazard model include proportional hazards, and additivity [34]. These assumptions were tested and fulfilled.

To test the robustness of the associations between steps and long-term sickness absence, a sensitivity analysis was performed, where participants who had experienced a pre-event of long-term sickness absence were excluded to test for reverse causation, i.e. that previous sickness absence was determining physical demands at work. In additional analyses, we also included pre-events of long-term sickness absence as a potential confounding variable. Pre-events of long-term sickness absence were identified through the DREAM register as events of long-term sickness absence 12 months prior to baseline. After exclusion of the participants with a pre-event, the crude and adjusted Cox proportional hazard models were re-run, and the HRs and 95% CI were then compared with results of the main analyses.

In another sensitivity analysis, we investigated if participants’ self-rated health, an assumed mediator (as illustrated in the DAG in Fig. 1A in the Additional file 1) changed the results. Self-rated health was assessed with the question: “How will you rate your overall health”, with the response categories “very good”, “good”, “fair”, “poor” or “very poor”.

Moreover, the presence of interaction was examined by adding a product term into the adjusted Cox proportional hazard models between the following variables: age and steps, sex and steps, job type and steps, smoking and steps and finally steps at work and steps at leisure. Interactions were tested in the total daily steps and the domain specific models, respectively. The overall interaction tested were indicated by the value of the likelihood ratio test (significance value set at less than 0.05).

## Results

Among the 2998 workers, 1390 (46.4%) filled out the baseline questionnaire and participated in a health check. Then, 1353 workers (45.1%) agreed to wear accelerometers for four consecutive days. Of these, 937 workers (31.3%) were eligible for inclusion in the analyses Fig. 1.

Of the 937 participants, a total of 192 participants (20.5%) experienced an event of long-term sickness absence during the four-years’ follow-up (Table 1). 52 participants (5.5%) had experienced a pre-event of long-term sickness absence in the 12 months prior to baseline. Slightly over half (55.1%) of the study population were men and the mean age at baseline was 44 years. The majority (83.6%) of the study population were blue-collar workers.

**Table 1:** Baseline characteristics of the study participants divided by quartiles of total daily steps

	**Quartile 1**	**Quartile 2**	**Quartile 3**	**Quartile 4**	**Total**	***p***
Total daily steps	< 9472	9472—12,496	12,497 – 16,473	> 16,473		< 0.001
Participants, n	234	235	234	234	937	
Steps work, M (SD)	4124 (1601)	6853 (1,819)	9014 (2,365)	13,499 (3,414)	8371 (4,186)	< 0.001
Steps leisure, M (SD)	3380 (1,366)	4177 (1,787)	5331 (2,186)	6681 (3,201)	4892 (2,560)	< 0.001
LTSA Event, n (%)	44 (18.8)	45 (19.1)	49 (20.9)	54 (23.1)	192 (20.5)	0.646
Male, n (%)	126 (53.8)	139 (59.1)	133 (56.8)	118 (50.4)	516 (55.1)	0.256
Age in years, M (SD)	45 (9.5)	44.7 (10.1)	44.9 (9.4)	44.6 (9.8)	44.9 (9.7)	0.841
Blue-collar worker, n (%)	138 (59.0)	204 (86.8)	216 (92.3)	225 (96.3)	783 (83.6)	< 0.001
Smoker^a^, (%)	58 (25.0)	81 (34.9)	71 (30.7)	67 (28.8)	277 (29.8)	0.129
Occ. lift/carrying^b^, n (%)						< 0.001
Almost all the time	17 (7.3)	23 (9.8)	28 (12.0)	29 (12.4)	97 (10.4)	
Approx. ¾ of the time	7 (3.0)	18 (7.7)	29 (12.4)	32 (13.7)	86 (9.2)	
Approx. ½ of the time	29 (12.4)	38 (16.2)	33 (14.2)	45 (19.2)	145 (15.5)	
Approx. ¼ of the time	38 (16.3)	57 (24.3)	60 (25.8)	62 (26.5)	217 (23.2)	
Rarely/very little	80 (34.3)	78 (33.2)	74 (31.8)	51 (21.8)	283 (30.3)	
Never	62 (26.6)	21 (8.9)	9 (3.9)	15 (6.4)	107 (11.4)	
Censored, n (%)	12 (5.1)	15 (6.4)	10 (4.3)	10 (4.3)	47 (5.0)	0.689
Pre-event, n (%)	9 (3.8)	13 (5.5)	13 (5.6)	17 (7.3)	52 (5.5)	0.456
Measured work days, M (SD)	3.0 (1.0)	2.7 (1.0)	2.9 (1.0)	2.9 (1.0)	2.9 (1.02)	0.032
Measured leisure days, M (SD)	2.6 (1.3)	2.4 (1.3)	2.5 (1.4)	2.6 (1.3)	2.6 (1.3)	0.297

*n* number of participants, *M* Mean, *SD* Standard Deviation, *LTSA* Long-term sickness absence, *Occ*. Occupational, *Approx*. approximately, *p* = *p*-value (level of significance set at < 0.005)

^a^nine missing values of smoking

^b^two missing values of occ. lifting/carrying

We found small differences between the participants across the four quartiles of total daily steps. Mean age was approximately evenly distributed between the quartiles. Events of long-term sickness absence increased with every quartile, from 44 events in Q1 to 54 events in Q4 quartile. Pre-events of long-term sickness absence in the year prior to baseline had occurred almost twice as frequently in Q4 (7.3%) compared with Q1 (3.8%). The percentage of blue-collar workers and duration of occupational lifting and carrying increased with every quartile. To investigate statistical differences between quartile groups, Chi-Square and ANOVA tests were conducted, with a *p*-value (*p*) less than 0.05 considered to be statistical significant. We found significant differences between groups in terms of total daily steps, steps at work and steps during leisure, job type, occupational lifting/carrying and measured work days (Table 1).

A statistically significant association was found between steps at work and long-term sickness absence (Table 2). In model 2, with adjustment for age, sex, smoking, job type and steps at leisure, HR was 1.040 (CI 95% 1.003–1.077) indicating that an individual, who took 1000 steps more at work, had a 4.0% higher risk of long-term sickness absence. Results of the analyses of steps at leisure and long-term sickness absence resulted in HRs just below 1, indicating a trend of a lower risk of long-term sickness absence with more steps during leisure. However, the results were not statistically significant. Results from the analyses of total daily steps and long-term sickness absence showed in both the crude and in the adjusted models a HR very close to 1, indicating no association between total daily steps and long-term sickness absence.

**Table 2:** Results of the crude and adjusted Cox proportional hazards models with Hazard Ratios (HR) of long-term sickness absence with 95% Confidence intervals (CI) per 1,000 steps at work, steps during leisure or total daily steps, respectively

	Work steps		Leisure Steps		Total daily steps	
	HR	(CI)	HR	(CI)	HR	(CI)
Crude model	1.036	(1.003–1.070)	0.983	(0.930–1.040)	1.020	(0.993–1.047)
Model 1	1.038	(1.005–1.072)	0.971	(0.916–1.029)	1.019	(0.992–1.046)
Model 2	1.040	(1.003–1.077)	0.965	(0.910–1.023)	1.016	(0.987–1.046)

*HR* Hazard Ratio. *CI *= 95% Confidence interval

Crude model: No adjustment (*n* = 937). Model 1: Adjusted for sex and age (*n* = 937). Model 2: Adjusted for age, sex, smoking and job type + analyses of work steps are adjusted for leisure steps, and analyses of leisure steps are adjusted for work steps (*n* = 928)

### Results of sensitivity analyses

Results of the Cox proportional hazard model analyzing steps and long-term sickness absence, including occupational lifting/carrying as a potential confounder, did not change the direction of the findings of the study (Table 3).

**Table 3:** Results of the Cox proportional hazards models adjusted for potential confounders age, sex, smoking, job type and occupational carrying/lifting duration (analyses of work steps are also adjusted for leisure steps and analyses of leisure steps are adjusted for work steps) with Hazard Ratios (HR) of risk of long-term sickness absence and 95% Confidence interval (CI) per 1,000 steps at work, steps during leisure or total daily steps respectively (*n* = 926)

	**Work steps**	**Leisure steps**	**Total daily steps**
	HR (CI)	HR (CI)	HR (CI)
Model 3	1.033 (0.996–1.071)	0.966 (0.910–1.026)	1.012 (0.983–1.043

*HR* Hazard Ratio. *CI* = 95% Confidence interval

Model 3: Adjusted for age, sex, smoking and job type + analyses of work steps are adjusted for leisure steps and analyses of leisure steps are adjusted for work steps + occupational lifting/carrying

When we excluded participants who had experienced pre-event of long-term sickness absence 12 month prior to baseline (*N* = 52) from the main analyses, results were consistent with the main findings (Table 4).

**Table 4:** Results of the crude and adjusted Cox proportional hazard models with Hazard Ratios (HR) of long-term sickness absence with 95% Confidence intervals (CI) per 1,000 steps at work, during leisure or total daily steps, respectively among the participants who had not experienced a pre-event of long-term sickness absence in the 12 month prior to baseline (*n* = 885)

	**Work steps**	**Leisure steps**	**Total daily steps**
	HR (CI)	HR (CI)	HR (CI)
Crude model	1.044 (1.009–1.081)	0.967 (0.908–1.029)	1.022 (0.993–1.052)
Model 1	1.046 (1.011–1.083)	0.953 (0.893–1.016)	0.977 (0.961–0.992)
Model 2	1.054 (1.015–1.094)	0.945 (0.885–1.010)	1.021 (0.944–1.807)

*HR* Hazard Ratio. *CI* = 95% Confidence interval. Crude model (no adjustment)

Model 1: Adjusted for sex and age. Model 2: Adjusted for age, sex, smoking and job type + analyses of work steps are adjusted for leisure steps and the analyses of leisure steps are also adjusted for work steps

Moreover, results also remained consistent after adjustment for pre-events (Results are presented in Table 1A in the Additional file 1). For 921 participants (98.3% of the sample) we had information about self-rated health. Further adjustment for this assumed mediator between steps and long-term sickness absence did not change the results substantially (Results are presented in Table 2A in the Additional file 1). None of the interaction terms tested in the models were significant (Results are presented in Tables 3A and 4A in the Additional file 1).

## Discussion

We investigated the association between accelerometer-measured steps walked at work and during leisure and register-based long-term sickness absence. We found that a higher number of steps at work were associated with a higher risk of long-term sickness absence. Steps during leisure and total daily steps were not clearly associated with long-term sickness absence.

Specifically, we found that every 1000 additional steps walked at work were significantly associated with 4% higher risk of long-term sickness absence. Conversely, we found a trend indicating that an increase of 1000 steps during leisure time resulted in a similarly lower risk of long-term sickness absence. While it was not possible to determine a statistically significant association between steps during leisure and long-term sickness absence, the strength of the association was comparable to steps at work, just in opposite direction. This result remained robust even with adjustments for potential confounders, such as sex, age, smoking, job type and steps at leisure.

None of the studies we have found to investigate steps and sickness absence, had information on steps taken at work versus during leisure time [5, 6, 16, 17]. Two previous studies found that walking during leisure was associated with a reduced risk of long-term sickness absence [5, 6], while one study found no significant association between walking while commuting to work and sickness absence [16], and another study found no significant association between walking at work and long-term sickness absence [17]. None of the studies measured total daily steps, and only one reported and analyzed domain-specific walking. Differences in measures of exposure and outcome, as well as methods in the various studies hamper comparison with the findings in our study. Thus, there is a need to confirm our results by conducting similar future studies investigating the association between technical-measured steps taken during work and during leisure, and prospective register-based information on sickness absence.

A previous study by Gupta et al. [12] found, that the amount of time spent on moderate to vigorous physical activity (MVPA) during leisure time was associated with lower risk of long-term sickness absence, while MVPA during work time was associated with higher risk of long-term sickness absence. However, they did not find any association between physical activity of lower intensities and long-term sickness absence [12]. In this study, we found a weaker association between the number of steps taken and long-term sickness absence. This could be because most of the steps taken are likely of lower intensity than MVPA. This suggests that the number of steps is somewhat important for the risk of sickness absence, but engaging in physical activity at higher intensities (MVPA) is particularly important for reducing the risk of long-term sickness absence.

The mechanisms of how steps at work may increase the risk of sickness absence are unknown. However, investigating the characteristics of steps taken at work as opposed to steps during leisure can give some indication. Compared with steps during leisure, workers may experience lower degree of control and influence on steps at work. Given that steps is a crucial physical activity for performing a number of work tasks, it can lead to the workers getting tired, strained or experiencing pain, without the possibility to adjust the amount or intensity of the steps taken and when to take breaks. Thus, steps taken at work might be monotonously performed, often at a low intensity level over several hours per day, without sufficient breaks and recovery time [15]. Further research is needed to better understand the potential mechanisms of how steps taken at work can increase sickness absence.

We expected that the number of steps during leisure would be associated with a lower risk of long-term sickness absence. However, we did not find a clear association between steps during leisure and sickness absence. One possible reason for our results may be differences between the study population of previous intervention studies and in the present study. Most intervention studies have been performed on adults with a very low total number of steps per day (an increased number of steps in leisure time among people with a very low number of steps at work, naturally provides better health). However, we also included individuals who had very high number of total daily steps, such as cleaners and manufactory workers (mean number steps at work was 8370 with a standard deviation of 4186). In the future, it may be important also to investigate the potential beneficial or harmful effect of steps during leisure for long-term sickness absence among different job groups with a higher variability in the number of steps walked at work.

We found no clear association between total daily steps and long-term sickness absence. This makes sense since steps at work indicated a harmful association with long-term sickness absence and steps during leisure indicated a potentially beneficial association. However, this result on total daily steps contrasts with a number of other studies that have examined daily number of steps and correlation with other health outcomes, such as all-cause mortality and cardio-vascular morbidity [1]. This difference can be due to either, as mentioned above, our population is different from these previous studies, or that illness from cardiovascular disease and death is very different from sickness absence. Especially, since sickness absence is a behavior related to one's state of health and work, and is influenced by many factors other than illness alone such as psychosocial determinants or physical job demands [35, 36]. Finally, our results emphasize the importance of dividing into domains when examining steps walked.

### Strength and limitations

A major strength of this study is that long-term sickness absence is based on data from the DREAM register, which gives a valid measurement of sickness absence [31]. Another major strength is that steps are objectively measured using accelerometers, which have shown high sensitivity and specificity, and superiority to self-reported measures of physical activity [18, 27, 37]. Also, the possibility to make domain-specific analyses is a considerable strength. Finally, we were able to take into account several possible covariates such as job type, smoking and occupational lifting/carrying, and show that the associations were independent from those covariates.

A limitation of the study is the observational study design, from which an inference about a causal relationship cannot be made.

The definition of long-terms sickness absence as 6 or more weeks might cause an underestimation of long-term sickness absence, since it misses shorter periods of sickness absence. The definition of 6 weeks is rather conservative, but the threshold was chosen as long-term sickness absence is a term affected by the political scene, causing the legislation (more specifically, when workplaces are getting reimbursed by the municipality) and definitions to have changed several times. However, the exclusion of shorter periods of sickness absence enabled this study to focus on more serious cases of sickness absence, and avoid the potential influence of minor illnesses.

In this study, the accelerometer is securely fastened to the participant’s thigh, which limits ability to remove and reattach it. Consequently, wear time is less of a concern compared with other studies that use accelerometers placed in belts on the hip or in a watch. Instead, the primary limitation in this study is the number of days of measurement. Since the average measured days was approximately 2.9 days at work and 2.6 days during leisure, which is below the recommended wearing time, the reliability of data on steps could be challenged [38, 39].

Another weakness to the study is that measurements of exposure and covariates were only conducted one time at baseline four years prior to follow-up. Participant’s daily number of steps, work tasks and work environment might have changed multiple times since baseline, and if there are non-random organizational changes, or changes in worker instructions at the workplace, it could affect the prevalence and timing of long-term sickness absence during follow-up.

Selection bias may also be of a concern in this study due to self-selection into the cohort. The recruitment of workplaces willing to allow employees to conduct all measurements during working hours, may have introduced selection bias among the workplaces, as it might be primarily workplaces with higher resources that choose to participate. This might cause an underestimation of the association between number of steps and long-term sickness absence risk. Lastly, a limitation is that job-type, a very crude variable, is the only indictor of socioeconomic status in this study. As the cohort data is based on a Danish context, generalizability of findings to other countries should be cautioned.

Previous advice regarding steps have been to just increase physical activity with number of steps as a central component of physical activity, no matter the domain [40]. Our results show that this kind of preventive advice, if implemented at work, might actually lead to an increased risk of long-term sickness absence. Our results indicate the need for better tailored advice depending on domain and type of work, and may suggest a potential of preventing long-term sickness absence by lowering number of steps during work among blue collar workers. Because workers in many jobs walk up to several thousand steps at work, a 4% higher long-term sickness absence risk per 1000 additional steps can be of great importance for prevention of long-term sickness absence among manual workers. Moreover, it can help to explain the high long-term sickness absence in job groups where workers spend much time on their feet (e.g., cleaners, eldercare workers).

Our study also shows that steps during leisure time alone do not seem to be related to lower risk of long-term sickness absence. Firstly, this points to, that advices should take domain of physical activity into consideration. Secondly, while walking many steps is good for the prevention of a number of diseases, the association with sickness absence is more complex. Based on the results of this study, we recommend future studies take domain-specific measurements, analyses and interpretations when investigating the association between number of steps and health outcome.

## Conclusion

This study suggests that a higher number of steps at work is associated with higher risk of long-term sickness absence. No statistically significant association was found for steps walked during leisure or total daily steps. These results emphasize the necessity to take domain of the steps into consideration, when investigating steps and health. Our findings should be verified by intervention studies, to make quantifiable guidelines on steps taken at work.

## Supplementary Information

**Additional file 1.**

## Data Availability

The fully anonymized data on prospective long-term sickness absence is available upon request from statistics Denmark (A Central Authority on Danish Statistics: https://dst.dk/da). The fully anonymized data from the baseline in NOMAD and DPHACTO from each participant involved in the analysis of this study are available in a Danish public repository (DPhacto: http://dda.dk/catalogue/28618?lang=en, NOMAD: http://dda.dk/catalogue/28617?lang=en).

## Abbreviations

Acc: Accelerometer
CI: Confidence intervals
DPhacto: The ‘Danish Physical activity cohort with objective measurements’
DREAM: Danish Register for Evaluation of Marginalization
HR: Hazard Ratio
LTSA: Long-term sickness absence
M: Mean
NOMAD: ‘New method for Objective Measurements of physical Activity in Daily living’
Occ.: Occupational
PODESA: ‘Physical wOrk DEmands and prospective register-based Sickness Absence study’
Q: Quartiles
SD: Standard deviation
