# Supplementary material for: Does it matter for health if steps are taken during work or leisure? A prospective accelerometer study using register-based long-term sickness absence
Source: Int J Behav Nutr Phys Act. 2023 Jun 9;20:69. doi: 10.1186/s12966-023-01468-4 (PMC10251587; doi:10.1186/s12966-023-01468-4)
Supplement: Supplementary file 1 — Additional file 1. [file 12966_2023_1468_MOESM1_ESM.docx]

**Additional file 1**

**Directed Acyclic Graphs**

A directed acyclic graph (DAG) of the association between number of steps and long-term sickness absence including all covariates and their associations is presented in Figure 1A.


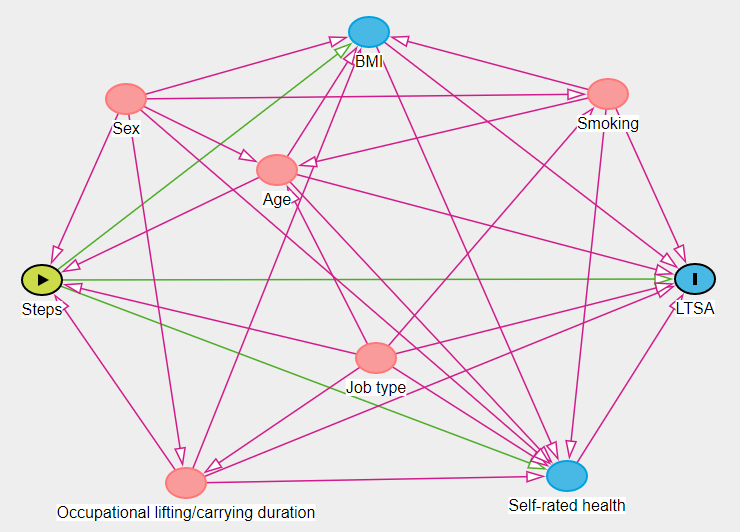


**Figure 1A.** Directed Acyclic Graph (DAG) Made with *DAGgitty* (40). LTSA=Long-term sickness absence, BMI=Body mass index.

The arrows in the DAG, representing associations between the variables, were placed based on the previous mentioned literature and on background knowledge of how the covariates were related. The blue variables represent mediators. Red variables indicate confounders while the red arrows represents biasing path. Green paths represent causal paths and the blue variables represent mediators (Except LTSA (long-term sickness absence, which is the outcome).

**Results of Cox proportional hazards models adjusted for pre-events of long-term sickness absence**

| **Table 1A** Results of the Cox proportional hazards models adjusted for pre-events of long-term sickness absence, with Hazard Ratios (HR) of long-term sickness absence with 95% Confidence intervals (CI) per 1,000 steps at work, steps at leisure or total daily steps, respectively | | | | | | |
| --- | --- | --- | --- | --- | --- | --- |
|  | Work steps | | Leisure Steps | | Total daily steps | |
|  | HR | (CI) | HR | (CI) | HR | (CI) |
| Crude model | 1.032 | (1.000-1.066) | 0.972 | (0.972-1.027) | 1.014 | (0.988-1.041) |
| Model 1 | 1.035 | (1.002-1.068) | 0.966 | (0.912-1.022) | 1.015 | (0.988-1.042) |
| Model 2 | 1.038 | (1.002-1.075) | 0.961 | (0.907-1.017) | 1.014 | (0.985-1.043) |

HR=Hazard Ratio. CI=95% Confidence interval.

Crude model: Adjusted for pre-event (*n* = 937). Model 1: Adjusted for sex, age and pre-event (*n* = 937). Model 2: Adjusted for age, sex, smoking, job type and pre-event + analyses of work steps are adjusted for leisure steps, and analyses of leisure steps are adjusted for work steps (*n* = 928).

**Results of Cox proportional hazards models adjusted for self-rated health**

| **Table 2A** Results of the Cox proportional hazards models adjusted for self-rated health, with Hazard Ratios (HR) of long-term sickness absence with 95% Confidence intervals (CI) per 1,000 steps at work, steps at leisure or total daily steps, respectively | | | | | | |
| --- | --- | --- | --- | --- | --- | --- |
|  | Work steps | | Leisure Steps | | Total daily steps | |
|  | HR | (CI) | HR | (CI) | HR | (CI) |
| Model 1 | 1.036 | (1.002-1.071) | 0.989 | (0.924-1.038) | 1.019 | (0.991-1.047) |
| Model 2 | 1.038 | (1.001-1.077) | 0.974 | (0.918-1.032) | 1.018 | (0.988-1.048) |

HR=Hazard Ratio. CI=95% Confidence interval.

Model 1: Adjusted for sex and age and self-rated health (*n* = 921). Model 2: Adjusted for age, sex, smoking and job type and self-rated health + analyses of work steps are adjusted for leisure steps, and analyses of leisure steps are adjusted for work steps (*n* = 921).

**Results of analysis of interactions between steps at work and confounding variables in Cox proportional hazards models**

| **Table 3A** Results of the interactions between step at work and confounding variables in the adjusted Cox proportional hazards models with Hazard Ratios (HR) of long-term sickness absence with 95% Confidence intervals (CI) per 1,000 steps at work | | |
| --- | --- | --- |
|  | HR | (CI) |
| Model 1 |  |  |
| Work steps x age | 1.001 | 0.998-1.005 |
| Work steps x sex | 1.006 | 0.942-1.073 |
| Model 2 |  |  |
| Work steps x age | 1.001 | 0.997-1.005 |
| Work steps x sex | 1.006 | 0.941-1.074 |
| Work steps x leisure steps | 1.003 | 0.992-1.015 |
| Work steps x smoking | 0.977 | 0.909-1.022 |
| Work steps x job type | 0.978 | 0.841-1.137 |

HR=Hazard Ratio. CI=95% Confidence interval.

Model 1: Adjusted for sex, age and work steps (*n* = 937). Model 2: Adjusted for age, sex, steps at leisure, steps at work, smoking, and job type (*n* = 928).

**Results of analysis of interactions between steps at leisure and confounding variables in Cox proportional hazards models**

| **Table 4A** Results of the interactions between step at leisure and confounding variables in the adjusted Cox proportional hazards models with Hazard Ratios (HR) of long-term sickness absence with 95% Confidence intervals (CI) per 1,000 steps at leisure | | |
| --- | --- | --- |
|  | HR | (CI) |
| Model 1 |  |  |
| Leisure steps x age | 1.005 | 0.998-1.011 |
| Leisure steps x sex | 0.990 | 0.881-1.111 |
| Model 2 |  |  |
| Leisure steps x age | 1.006 | 1.000-1.013 |
| Leisure steps x sex | 0.998 | 0.888-1.121 |
| Leisure steps x work steps | 1.003 | 0.992-1.015 |
| Work steps x smoking | 0.984 | 0.863-1.121 |
| Work steps x job type | 0.953 | 0.774-1.173 |

HR=Hazard Ratio. CI=95% Confidence interval.

Model 1: Adjusted for sex, age and leisure steps (*n* = 937). Model 2: Adjusted for age, sex, steps at leisure, steps at work, smoking, and job type (*n* = 928).
